# Likelihood of Lung Cancer Screening by Poor Health Status and Race and Ethnicity in US Adults, 2017 to 2020

**DOI:** 10.1001/jamanetworkopen.2022.5318

**Published:** 2022-03-31

**Authors:** Alison S. Rustagi, Amy L. Byers, Salomeh Keyhani

**Affiliations:** 1 Medical Service, San Francisco Veterans Affairs Health Care System, San Francisco, California; 2 Department of Medicine, University of California, San Francisco; 3 Research Service, San Francisco Veterans Affairs Health Care System, San Francisco, California; 4 Department of Psychiatry and Behavioral Sciences, University of California, San Francisco; 5 Weill Institute for Neurosciences, University of California, San Francisco

## Abstract

**Importance:**

Lung cancer screening (LCS) via low-dose chest computed tomography can prevent mortality through surgical resection of early-stage cancers, but it is unknown whether poor health is associated with screening. Though LCS may be associated with better outcomes for non-Hispanic Black individuals, it is unknown whether racial or ethnic disparities exist in LCS use.

**Objective:**

To determine whether health status is associated with LCS and whether racial or ethnic disparities are associated with LCS independently of health status.

**Design, Setting, and Participants:**

This cross-sectional, population-based study of community-dwelling US adults used data from Behavioral Risk Factor Surveillance System annual surveys, 2017 to 2020. Participants were aged 55 to 79 years, with a less than 30 pack-year smoking history, and were current smokers or those who quit within 15 years. Data were analyzed from August to November 2021.

**Exposures:**

Self-reported health status and race and ethnicity.

**Main Outcomes and Measures:**

Self-reported LCS in the last 12 months.

**Results:**

Of 14 550 individuals (7802 men [55.5%]; 7527 [55.0%] aged 65-79 years [percentages are weighted]), representing 3.68 million US residents, 17.0% (95% CI, 15.1%-18.9%) reported undergoing LCS. The prevalence of LCS was lower among non-Hispanic Black than non-Hispanic White individuals but not to a significant degree (12.0% [95% CI, 4.3%-19.7%] vs 17.5% [95% CI, 15.6%-19.5%]; *P* = .57). Health status was associated with LCS: 468 individuals in poor health vs 96 individuals in excellent health reported LCS (25.2% [95% CI, 20.6%-29.9%] vs 7.6% [95% CI, 5.0%-10.3%]; *P* < .001), and those with difficulty climbing stairs were more likely to report LCS than those without this functional limitation. Adjusting for sociodemographic factors, functional status, and comorbidities, self-rated health status remained associated with LCS (adjusted odds ratio, 1.19 per each 1-step decline in health; 95% CI, 1.03-1.38), and non-Hispanic Black individuals were 53% less likely to report LCS than non-Hispanic White individuals (adjusted odds ratio, 0.47; 95% CI, 0.24-0.90). Results were robust in sensitivity analyses in which health was alternatively quantified as number of comorbidities.

**Conclusions and Relevance:**

LCS in the US is more common among those who may be less likely to benefit from screening because of poor underlying health. Furthermore, racial or ethnic disparities were evident after accounting for health status, with non-Hispanic Black individuals nearly half as likely as non-Hispanic White individuals to report LCS despite the potential for greater benefit of screening this population.

## Key Points

**Question:**

Is lung cancer screening reaching individuals who can gain the most from it?

**Findings:**

In this cross-sectional study of 14 550 US individuals, patients with worse self-rated health or more medical conditions, which can limit the benefit of early detection of lung cancer, were more likely than individuals with better health status to report lung cancer screening. Adjusting for health status, non-Hispanic Black individuals were 53% less likely to report screening than non-Hispanic White individuals.

**Meaning:**

These findings suggest that individuals in poor health are more likely than those with good health status to undergo lung cancer screening, which can lessen its potential mortality benefit, and non-Hispanic Black individuals are less likely to undergo lung cancer screening despite its likely greater benefit in this group.

## Teaser

This cross-sectional, population-based study examines rates of lung cancer screening to evaluate whether health status and racial and ethnic disparities are associated with rates of screening.

## Introduction

Lung cancer is the leading cause of cancer deaths in the US.^1^ From 2013 to 2021, the US Preventive Services Task Force recommended lung cancer screening (LCS) via low-dose computed tomography for adults aged 55 to 80 years with a greater than 30 pack-year history who are current smokers or quit within 15 years.^2^ The US Preventive Services Task Force additionally highlights the need to screen only those willing and able to undergo lung surgery,^2,3^ and all national guidelines recommend against screening those with poor baseline health status, whether quantified as less than 10 years’ life expectancy (American College of Chest Physicians),^4^ “life limiting comorbid conditions” (American Cancer Society),^5^ or “as long as patient functional status and comorbidity allow consideration for curative intent therapy” (National Comprehensive Cancer Network).^6^ Although national guidelines are consistent in recommending that baseline health should be considered prior to screening, how to define poor health is unclear, and the American Thoracic Society recognizes that there is an urgent need for more research in this area.^7^

The mortality benefit of LCS is thought to be largely achieved through surgical resection of early-stage cancers to achieve a cure, or improve survival after adjuvant therapies.^8,9^ Two pivotal randomized clinical trials were powered to detect a mortality benefit of LCS: the National Lung Screening Trial (NLST)^8^ in the US and the Nederlands-Leuvens Longkanker Screenings Onderzoek (NELSON) trial^10^ in Europe. The NLST excluded those who were not healthy enough to be considered surgical candidates per individualized clinical evaluation, and the NELSON trial excluded individuals who rated their health as moderate or bad or could not climb 2 flights of stairs, as a proxy of health.^10^ Self-rated health and ability to climb stairs are thus key metrics that are broadly available and can provide a point of comparison to general population data.

However, little is known about whether individuals in fairly good health are more or less likely to be screened than those in poor health. The general US population of screen-eligible individuals has a higher mortality rate than NLST participants.^11^ In fact, those in poor health may be paradoxically more likely to undergo LCS.^12^ Individuals with chronic health conditions are more likely to engage in regular health visits, which has been associated with LCS.^13^ This raises concern that increased mortality associated with lung surgery and/or competing causes of death could attenuate the 16% mortality benefit associated with LCS.^14^

Another important consideration is that LCS may have better outcomes among non-Hispanic Black individuals. In secondary analyses of NLST trial data, non-Hispanic Black participants experienced a greater reduction in lung-cancer specific mortality (hazard ratio [HR] 0.61 [95% CI, 0.37-1.01] vs 0.86 [95% CI, 0.75-0.98] among non-Hispanic White participants) and all-cause mortality (HR, 0.81 [95% CI, 0.65-1.00] vs 0.95 [95% CI, 0.89-1.02]; *P* < .05).^15^ If LCS is less likely to reach non-Hispanic Black individuals, then racial disparities could further diminish the actual benefit of LCS by missing this important group. Racial disparities have been observed in single centers in Rhode Island,^16^ Pennsylvania,^17^ Indiana,^18^ and North Carolina,^19^ but population-level analyses have failed to show LCS disparities by race and ethnicity.^13,20,21,22,23^ However, poor self-rated health and all-cause mortality are still associated with race and ethnicity in the US,^24,25^ with non-Hispanic Black individuals experiencing a 20% higher all-cause mortality than non-Hispanic White individuals,^26^ in large part due to structural racism.^27^ If poor health is associated with increased screening, and individuals from historically minoritized racial and ethnic groups are more likely to be in poor health, then racial or ethnic disparities could be obscured unless comparisons account for health status. Indeed, prior population-based analyses were limited by small samples of individuals who were not non-Hispanic White, and did not account for health status.

Using the data from the Behavioral Risk Factor Surveillance System (BRFSS), we examined the association of health status with undergoing LCS among US adults. We also examined differences in undergoing LCS by race and ethnicity after adjusting for health status.

## Methods

### Data Source and Population

Data for this cross-sectional, population-based study were drawn from the 2017 to 2020 data sets of BRFSS, an annual telephone survey of noninstitutionalized US adults conducted by the US Centers for Disease Control and Prevention.^28^ BRFSS conducts more than 400 000 interviews with residents of all 50 states, the District of Columbia, and 3 territories to ascertain behaviors associated with health and health service utilization. Core questions are asked of all respondents; states may opt to add additional topic-specific modules. LCS eligibility and use are included in an optional module that was conducted in 11 states in 2017 (Florida, Georgia, Kansas, Kentucky, Maine, Maryland, Missouri, Nevada, Oklahoma, Vermont, and Wyoming),^29^ 8 states in 2018 (Delaware, Maryland, Maine, New Jersey, Oklahoma, South Dakota, Texas, and West Virginia),^30^ 20 states in 2019 (Arizona, Idaho, Kansas, Kentucky, Maine, Maryland, Minnesota, Missouri, Montana, Nebraska, North Carolina, North Dakota, Oklahoma, Pennsylvania, Rhode Island, South Carolina, Utah, Vermont, West Virginia, and Wisconsin),^31^ and 5 states in 2020 (Delaware, Maine, New Jersey, North Dakota, and South Dakota),^32^ in total representing 28 unique states. We identified US adults eligible for LCS by age and smoking history according to 2013 to 2021 USPTF guidelines and limited the sample to individuals aged 55 to 79 years, with a greater than 30 pack-year smoking history, and current smoker or former smoker who quit within 15 years prior. Exclusion criteria were personal history of lung cancer and age less than 55 or equal to or greater than 80 years. Individuals with missing values for these variables were excluded from the analysis; among 155 562 respondents aged 55 to 79 years with no personal history of lung cancer, 92.7% had complete data on tobacco history to determine LCS eligibility.

This study used publicly available, deidentified data and, thus, was exempt from institutional review board review and the need for informed consent, in accordance with 45 CFR §46. Reporting conformed to the Strengthening the Reporting of Observational Studies in Epidemiology (STROBE) reporting guideline for cross-sectional studies.^33^

### Exposures

The primary exposures were self-rated health status and race and ethnicity. Health status was ascertained by the question, “Would you say that in general your health is Excellent, Very Good, Good, Fair, Poor?” In addition to this primary exposure, we ascertained the distribution of LCS by 3 functional limitations and 7 comorbidities: chronic kidney disease, arthritis, chronic obstructive pulmonary disease (COPD), asthma, vascular disease (history of myocardial infarction or stroke), diabetes, and personal history of cancer other than skin cancer. Each comorbidity was assessed with the question, “Has a doctor, or nurse, or other health professional ever told you that you had any of the following?” Functional limitations (no, yes) were ascertained by the following 3 questions: (1) “Do you have serious difficulty walking or climbing stairs?”; (2) “Do you have difficulty dressing or bathing?”; (3) and “Because of a physical, mental, or emotional condition, do you have difficulty doing errands alone such as visiting a doctor’s office or shopping?” Race and ethnicity were self-reported with the following survey categories: White non-Hispanic, Black or African American non-Hispanic, American Indian or Alaska Native non-Hispanic, Asian non-Hispanic, Native Hawaiian or other Pacific Islander non-Hispanic, other race non-Hispanic, multiracial non-Hispanic, and Hispanic.

### Outcome

The primary outcome was self-reported LCS, ascertained by the question, “In the last 12 months, did you have a CT or CAT [computed tomography] scan?” A respondent was counted as screened if they chose “Yes, to check for lung cancer.” Of those eligible for LCS, 1.9% had unknown screening status.

### Covariates

We chose covariates a priori on the basis of prior research. In general, cancer screening tests are associated with socioeconomic status and smoking behavior,^34^ which holds true for LCS.^12^ Correlates of LCS also include health care access,^23^ COPD, prior cancer,^13,22,35^ state of residence,^36^ receipt of prior vaccination,^12^ demographic characteristics (eg, marital status and employment), and smoking history in pack-years.^12^

To account for socioeconomic status and demographics, we extracted data on age (in 5-year age categories), sex (male and female), race and ethnicity (aforementioned categories), marital status (married, divorced, widowed, separated, never married, or unmarried couple), and educational attainment (never attended school or only kindergarten, elementary or middle school, some high school, high school graduate, some college, and college graduate or more). To describe health factors, we captured body mass index categories (<18.5, 18.5 to <25, 25 to <30, and ≥30, calculated as weight in kilograms divided by height in meters squared), smoking pack-year history (in quartiles), health insurance status (any vs none), receipt of influenza vaccine in prior 12 months (no vs yes), and difficulty paying for medical care (no vs yes), which was ascertained with the question: “Was there a time in the past 12 months when you needed to see a doctor but could not because of cost?”

### Statistical Analysis

We conducted univariable analyses for variables of interest, calculating *P* values from *t* tests, χ^2^ tests, or linear tests for trend as appropriate to evaluate differences in LCS prevalence. We used multivariable logistic regression to examine the association between LCS and self-rated health status and LCS and race and ethnicity. We created a model that included both self-rated health status and race and ethnicity to isolate the association between each of these variables and LCS. Finally, for the main analysis, we constructed a model with all the aforementioned variables plus functional limitations and comorbidities as covariates. We assessed multicollinearity using the variance inflation factor, with a conservative threshold of 5 indicating collinearity.^37^

Self-rated health status was modeled as an ordered categorical variable. Race and ethnicity were included in the models as a 3-category variable (non-Hispanic Black, non-Hispanic White, or other) for model stability, as the unweighted numbers of screened individuals in racial and ethnic subgroups other than non-Hispanic White and non-Hispanic Black were small. All covariates with more than 2 categories were included as indicator variables in regression models to allow model flexibility. All tests were 2-sided. *P* < .05 was considered significant. Weights followed standardized protocols from the US Centers for Disease Control and Prevention, accounting for complex survey design.

In sensitivity analyses, we replicated the aforementioned models but substituted the total number of chronic health conditions (0, 1, 2, or ≥3) as the primary exposure in place of self-rated health status. We also calculated the prevalence of comorbidities across categories of self-rated health status. All analyses were done in Stata version 13.1 (StataCorp). Data were analyzed from August 2021 to November 2021.

## Results

Among 14 828 respondents who were eligible for LCS according to age and smoking history, 14 550 (7802 men [55.5%]; 7527 [55.0%] aged 65-79 years; 12 955 [87.5%] non-Hispanic White [percentages are weighted]) had complete data on undergoing LCS. These individuals represented an underlying population of 3.68 million LCS-eligible US residents, from a sample representing 119 million US residents in total. Of eligible individuals, 17.0% (95% CI, 15.1%-18.9%) reported undergoing LCS.

### Weighted Prevalence of LCS by Demographic Factors

We observed significant differences by demographic factors (Table 1). LCS was associated with older age, with a screening rate of 19.2% (1338 individuals; 95% CI, 16.5%-22.0%) among those aged 65 to 79 years vs 15.2% (910 individuals; 95% CI, 12.5%-17.8%) among those 55 to 64 years (*P* = .04). Screening was less likely among those with difficulty paying for medical care (200 individuals who had difficulty paying for health care; 10.1% [95% CI, 7.4%-12.8%] vs 2041 individuals who did not; 17.9% [95% CI, 15.8%-20.1%]; *P* < .001). Sex, marital status, education, and body mass index were not significantly associated with LCS.

**Table 1.** Prevalence of Lung Cancer Screening by Demographic and Health Characteristics, Among Individuals Representing 3.68 Million Screen-Eligible US Individuals, 2017-2020

Variable	Total, unweighted No. (N = 14 550)	Underwent screening, unweighted No. (N = 2248)	Underwent screening, weighted % (95% CI)	*P* value	Missing^a^
Self-rated general health					
Excellent	846	96	7.6 (5.0-10.3)	<.001	49
Very good	3195	365	12.8 (7.8-17.7)		
Good	4966	670	15.4 (12.4-18.5)		
Fair	3488	641	20.6 (16.6-24.7)		
Poor	2006	468	25.2 (20.6-29.9)		
Race and ethnicity					
Non-Hispanic Black	446	58	12.0 (4.3-19.7)		
Non-Hispanic White	12 955	2011	17.5 (15.6-19.5)	.57	219
Other^b^	930	139	15.0 (3.5-26.5)		
Age, y					
55-64	7023	910	15.2 (12.5-17.8)	.04	0
65-79	7527	1338	19.2 (16.5-22.0)		
Sex					
Male	7802	1191	16.0 (13.7-18.2)	.28	5
Female	6743	1055	18.2 (14.9-21.4)		
Marital status					
Not married	8053	1269	18.2 (15.2-21.3)	.22	60
Married	6437	970	15.8 (13.5-18.1)		
Educational attainment					
High school diploma or less	7125	1086	17.2 (14.5-19.9)	.81	26
Some college or more	7399	1158	16.7 (14.0-19.4)		
Difficulty paying for medical care					
No	12 849	2041	17.9 (15.8-20.1)	<.001	38
Yes	1663	200	10.1 (7.4-12.8)		
Body mass index^c^					
<18.5	338	77	19.3 (10.5-28.2)	.42	579
18.5 to <25	3946	659	17.8 (14.4-21.1)		
25 to <30	4878	696	17.2 (13.2-21.2)		
≥30	4809	738	16.2 (13.5-19.0)		
Smoking history, pack-years^d^					
Quartile 1: 30-39	3729	427	12.9 (8.8-17.1)	.003	0
Quartile 2: 39-45	3633	527	15.1 (12.3-18.0)		
Quartile 3: 45-58.75	3756	668	19.7 (15.8-23.6)		
Quartile 4: >58.75	3432	626	21.1 (16.9-25.4)		
Difficulty walking or climbing stairs					
No	9363	1291	15.4 (13.1-17.8)	.04	60
Yes	5127	947	19.7 (16.4-23.1)		
Difficulty dressing or bathing					
No	13 090	1947	16.3 (14.3-18.3)	.06	27
Yes	1433	296	23.2 (16.4-30.0)		
Difficulty doing errands alone					
No	12 482	1834	16.2 (14.1-18.3)	.02	55
Yes	2013	405	22.3 (17.6-27.0)		
Chronic kidney disease					
No	13 593	2072	16.7 (14.7-18.7)	.38	58
Yes	899	161	20.5 (12.3-28.7)		
Arthritis					
No	7038	958	15.4 (12.6-18.2)	.09	88
Yes	7424	1279	18.7 (16.1-21.4)		
Chronic obstructive pulmonary disease					
No	9241	996	11.2 (9.0-13.4)	<.001	49
Yes	5260	1248	27.6 (24.1-31.1)		
Asthma					
No	12 679	1849	16.2 (14.2-18.2)	.09	136
Yes	1735	368	21.9 (15.7-28.0)		
Vascular disease^e^					
No	10 617	1518	16.4 (14.1-18.8)	.31	29
Yes	3904	723	18.5 (15.3-21.6)		
Diabetes					
No	11 329	1720	15.7 (13.6-17.8)	.01	23
Yes	3198	525	21.7 (17.4-25.9)		
Personal history of cancer^f^					
No	12 317	1618	14.7 (12.6-16.7)	<.001	50
Yes	2183	615	31.2 (26.1-36.3)		
No. of comorbidities^g^					
0	3354	316	11.0 (6.8-15.2)	<.001	0
1	4434	576	13.8 (10.8-16.8)		
2	3449	634	19.7 (16.4-23.1)		
≥3	3313	722	25.4 (20.8-29.9)		

^a^ Individuals with missing values were excluded from the analysis. For all categories, less than 5% of data were missing.

^b^ Other race is defined as American Indian or Alaska Native non-Hispanic, Asian non-Hispanic, Native Hawaiian or other Pacific Islander non-Hispanic, other race non-Hispanic, multiracial non-Hispanic, or Hispanic.

^c^ Body mass index is calculated as weight in kilograms divided by height in meters squared.

^d^ Smoking history is defined as mean number of packs smoked per day, multiplied by duration of smoking in years.

^e^ Vascular disease is defined as prior myocardial infarction, coronary heart disease, cerebrovascular accident.

^f^ Personal history of cancer excludes skin cancer. Individuals with a personal history of lung cancer were excluded from the analysis.

^g^ Comorbidities are defined as the following: chronic kidney disease, arthritis, chronic obstructive pulmonary disease, asthma, vascular disease, diabetes, or personal history of cancer other than skin cancer.

### Weighted Prevalence of LCS by Self-rated Health Status and Race and Ethnicity

Worsening self-rated health status was associated with screening, as seen in Table 1: screening prevalence was 25.2% (95% CI, 20.6%-29.9%) among those in poor health vs 7.6% (95% CI, 5.0%-10.3%) among those in excellent health (*P* < .001 for trend). Overall, 48.5% (95% CI, 42.2%-54.9%) of those screened were in fair or poor health. Unadjusted rates of screening increased between successively poorer health categories in an approximately linear manner (Figure, panel A). Non-Hispanic Black individuals and those of other races and ethnicities reported lower rates of screening compared with non-Hispanic White individuals, though these differences were not significant (12.0% [95% CI, 4.3%-19.7%] vs 17.5% [95% CI, 15.6%-19.5%]; *P* = .57) (Table 1).

**Figure.**
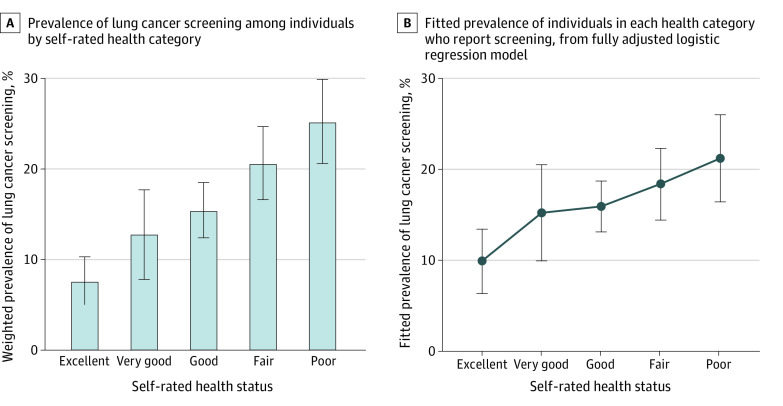
Prevalence of Lung Cancer Screening by Self-reported Health Status, Among 14 550 Individuals Representing 3.68 Million Screen-Eligible US Individuals, 2017-2020 Error bars indicate 95% CIs.

### Variation in LCS by Smoking History, Functional Status, and Comorbidities

LCS increased with increasingly heavy smoking history, with a screening rate of 12.9% (427 individuals; 95% CI, 8.8%-17.1%) among those in the lowest quartile of pack-year history vs 21.1% (626 individuals; 95% CI, 16.9%-25.4%) in the highest quartile (*P* = .003). LCS was more prevalent among those with each functional limitation: for example, among those with difficulty walking or climbing stairs, 19.7% (947 individuals; 95% CI, 16.4%-23.1%) reported being screened compared with only 15.4% (1291 individuals; 95% CI, 13.1%-17.8%) of those without this limitation (*P* = .04). Overall, 43.6% (1012 individuals; 95% CI, 37.4%-50.0%) of those screened reported at least 1 functional limitation. Screening was also associated with number of comorbidities, increasing from 11.0% (316 individuals; 95% CI, 6.8%-15.2%) of those with no known chronic health conditions, to 25.4% (722 individuals; 95% CI, 20.8%-29.9%) of those with 3 or more (*P* < .001). Overall, 33.9% (722 individuals; 95% CI, 28.1%-40.3%) of those screened had at least 3 chronic health conditions. Those with COPD, diabetes, or a personal history of cancer other than skin cancer were significantly more likely to report screening; for example, 27.6% (1248 individuals; 95% CI, 24.1%-31.1%) of those with COPD had been screened, compared with 11.2% (996 individuals; 95% CI, 9.0%-13.4%) of those without COPD (*P* < .001).

### Odds Ratios of Demographic and Health Characteristics With LCS

In a logistic regression model adjusted for demographics and health behaviors associated with screening likelihood (age, sex, marital status, body mass index, education, smoking history in pack-years, insurance status, receipt of influenza vaccine in prior 12 months, or difficulty paying for medical care), self-rated health was linearly associated with LCS: each 1-step decline in health status, such as from good to fair health, was associated with a 36% (95% CI, 20%-55%) higher likelihood of LCS (Table 2, model 1). There was no significant association between race and ethnicity and LCS in an unadjusted model or in a model adjusted for the demographics and health behaviors but not health status (model 2). In a maximally adjusted model that included specific functional limitations and diagnoses, as well as self-rated health status and race and ethnicity (model 3), the association between self-rated health status and LCS was attenuated in magnitude but remained significant (adjusted odds ratio [aOR], 1.19; 95% CI, 1.03-1.38). This association was approximately linear across all categories of self-rated health (Figure, panel B). However, the association between race and ethnicity and LCS was more precise in this model, as shown in Table 2, with non-Hispanic Black individuals 53% less likely to report LCS than non-Hispanic White individuals (aOR, 0.47; 95% CI, 0.24-0.90). Those who were not non-Hispanic Black or non-Hispanic White had similar likelihood of LCS as non-Hispanic White individuals, although precision was poor (aOR, 1.03; 95% CI, 0.35-3.01).

**Table 2.** ORs of the Associations Between Lung Cancer Screening, Self-rated Health Status, and Race and Ethnicity Among Individuals Representing 3.68 Million Screen-Eligible US Individuals, 2017-2020

Variable^d^	Unadjusted		Model 1^a^		Model 2^b^		Model 3^c^	
	OR (95% CI)	*P* value	aOR (95% CI)	*P* value	aOR (95% CI)	*P* value	aOR (95% CI)	*P* value
General health	1.36 (1.20-1.54)	<.001	1.36 (1.20-1.55)	<.001	NA	NA	1.19 (1.03-1.38)	.02
Race and ethnicity								
Non-Hispanic Black	0.64 (0.31-1.35)	.24	NA	NA	0.60 (0.30-1.21)	.15	0.47 (0.24-0.90)	.02
Non-Hispanic White	1 [Reference]	NA	NA	NA	1 [Reference]	NA	1 [Reference]	NA
Other^e^	0.83 (0.33-2.07)	.69	NA	NA	1.02 (0.41-2.53)	.96	1.03 (0.35-3.01)	.96
Difficulty walking or climbing stairs	1.35 (1.02-1.78)	.04	NA	NA	NA	NA	0.88 (0.63-1.24)	.46
Difficulty dressing or bathing	1.56 (1.03-2.34)	.03	NA	NA	NA	NA	1.13 (0.70-1.82)	.61
Difficulty doing errands alone	1.49 (1.09-2.040	.01	NA	NA	NA	NA	1.11 (0.72-1.69)	.64
Chronic kidney disease	1.28 (0.76-2.16)	.35	NA	NA	NA	NA	0.95 (0.58-1.58)	.86
Arthritis	1.27 (0.96-1.67)	.09	NA	NA	NA	NA	0.92 (0.69-1.22)	.56
Chronic obstructive pulmonary disease	3.02 (2.29-3.99)	<.001	NA	NA	NA	NA	2.42 (1.81-3.24)	<.001
Asthma	1.45 (0.98-2.13)	.06	NA	NA	NA	NA	0.97 (0.65-1.43)	.87
Vascular disease	1.15 (0.88-1.51)	.31	NA	NA	NA	NA	0.79 (0.59-1.05)	.11
Diabetes	1.49 (1.10-2.01)	.01	NA	NA	NA	NA	1.41 (1.04-1.92)	.03
Personal history of cancer	2.63 (1.97-3.52)	<.001	NA	NA	NA	NA	2.35 (1.76-3.14)	<.001

Abbreviations: aOR, adjusted odds ratio; NA, not applicable; OR, odds ratio.

^a^ Model 1 included the following covariates: age (5-year categories), sex (male or female), marital status (married, divorced, widowed, separated, never married, or part of an unmarried couple), body mass index (<18.5, 18.5 to <25, 25 to <30, or ≥30; body mass index is calculated as weight in kilograms divided by height in meters squared), educational attainment (never attended school or only kindergarten, elementary school, some high school, high school graduate, some college, or college graduate or more), smoking-history in pack-years (quartiles), insurance status (any vs none), receipt of influenza vaccine in prior 12 months (no vs yes), and difficulty paying for medical care (no vs yes). Exposure variable was self-rated health status.

^b^ Model 2 included the same covariates as model 1. Exposure variable was race and ethnicity.

^c^ Model 3 included the same covariates as model 1 and the listed functional limitations and specific diagnoses. Exposure variables were self-rated health status and race and ethnicity.

^d^ For binary variables (eg, difficulty walking or climbing stairs), ORs shown are relative to individuals without the listed limitation/diagnosis. General health was rated on a scale of excellent, very good, good, fair, or poor. OR is per 1-unit decline in self-rated health status (eg, from good to fair health). Vascular disease is defined as prior myocardial infarction, coronary heart disease, cerebrovascular accident. Personal history of cancer excludes skin cancer. Individuals with a personal history of lung cancer were excluded from the analysis.

^e^ Other race is defined as American Indian or Alaska Native non-Hispanic, Asian non-Hispanic, Native Hawaiian or other Pacific Islander non-Hispanic, other race non-Hispanic, multiracial non-Hispanic, or Hispanic.

No substantial collinearity between variables in model 3 was identified, with variance inflation factors for self-rated health status, difficulty walking, difficulty dressing, difficulty running errands alone, chronic kidney disease, arthritis, COPD, asthma, vascular disease, diabetes, and personal history of cancer ranging from 1.03 to 1.59, well below the accepted threshold of 5.^37^ In this model, COPD and personal cancer history persisted as independent factors significantly associated with LCS (COPD: aOR, 2.42 [95% CI, 1.81-3.24]; personal cancer history: aOR 2.35 [95% CI, 1.76-3.14]). In an exploratory analysis to test whether the observed association between self-rated health status and LCS were mediated by physician visit history, we included a variable capturing most recent physician check-up (less than 1 year, 1 to less than 2 years, 2 to less than 5 years, 5 or more years, or never) in model 3; the association between LCS and self-rated health did not change in magnitude or significance (aOR, 1.19; 95% CI, 1.02-1.37). In another exploratory analysis, no deviations from a linear association between self-rated health status and LCS was noted; when indicator variables for each health status category were included in model 3, in addition to the ordered categorical variable, none had significant coefficients (*P* values from .31 to .81).

### Sensitivity Analyses

In sensitivity analyses in which the primary exposure variable of self-rated health was replaced by number of comorbidities (0, 1, 2, or ≥3), the number of comorbidities was significantly associated with LCS. Individuals with 3 or more comorbidities were nearly 3 times as likely to undergo LCS as those with none of the ascertained health conditions (aOR, 2.75; 95% CI, 1.68-4.50) (Table 3). This association persisted after adjustment for demographic and health characteristics (model 4) and further adjustment for race and ethnicity (model 5). Worsening self-rated health was associated with increasing prevalence of each functional limitation and diagnosis (eFigure in the Supplement).

**Table 3.** ORs of the Associations Between Lung Cancer Screening, Number of Comorbidities, and Race and Ethnicity Among Individuals Representing 3.68 Million Screen-Eligible US Individuals, 2017-2020

Variable	Unadjusted		Model 4^a^		Model 5^b^	
	OR 95% CI	*P* value	aOR 95% CI	*P* value	aOR 95% CI	*P* value
No. of comorbidities^c^						
0	1 [Reference]	NA	1 [Reference]	NA	1 [Reference]	NA
1	1.30 (0.79-2.14)	.31	1.20 (0.74-1.94)	.46	1.19 (0.74-1.92)	.47
2	1.99 (1.23-3.21)	.01	1.81 (1.14-2.88)	.01	1.77 (1.12-2.81)	.02
≥3	2.75 (1.68-4.50)	<.001	2.47 (1.53-3.98)	<.001	2.38 (1.48-3.83)	<.001
Race and ethnicity						
Non-Hispanic Black	NA	NA	NA	NA	0.61 (0.30-1.28)	.20
Non-Hispanic White	NA	NA	NA	NA	1 [Reference]	NA
Other^d^	NA	NA	NA	NA	1.01 (0.39-2.61)	.99

Abbreviations: aOR, adjusted odds ratio; NA, not applicable; OR, odds ratio.

^a^ Model 4 included the following covariates: age (5-year categories), sex (male vs female), marital status (married, divorced, widowed, separated, never married, or part of an unmarried couple), body mass index (<18.5, 18.5 to <25, 25 to <30, or ≥30; body mass index is calculated as weight in kilograms divided by height in meters squared), educational attainment (never attended school or only kindergarten, elementary school, some high school, high school graduate, some college, or college graduate or more), smoking-history in pack-years (quartiles), insurance status (any vs none), receipt of influenza vaccine in prior 12 months (no vs yes), and difficulty paying for medical care (no vs yes). Exposure variable was number of comorbidities.

^b^ Model 5 included the same covariates as model 4. Exposure variables were number of comorbidities and race and ethnicity.

^c^ Comorbidities are defined as the following: chronic kidney disease, arthritis, chronic obstructive pulmonary disease, asthma, vascular disease, diabetes, personal history of cancer other than skin cancer.

^d^ Other race is defined as American Indian or Alaska Native non-Hispanic, Asian non-Hispanic, Native Hawaiian or other Pacific Islander non-Hispanic, other race non-Hispanic, multiracial non-Hispanic, or Hispanic.

## Discussion

In this cross-sectional study using population-based data from 28 states, LCS uptake differed considerably by self-rated health status, with individuals in poorer health more likely to report screening. Although overall screening rates remain low, nearly one-half of screened individuals rated their health as fair or poor, more than 40% had serious functional limitations, and one-third reported 3 or more comorbidities. Furthermore, racial and ethnic disparities were evident, with non-Hispanic Black individuals nearly 50% less likely than non-Hispanic White individuals to report screening after accounting for health status. This study suggests that, as implemented, the benefit of LCS may differ from that seen in randomized clinical trials, which were exclusively conducted in those healthy enough to undergo lung surgery and suggested greater benefit for non-Hispanic Black participants.

These results are concerning, as the long-term benefits of screening those with frail health are unknown; these individuals were excluded from the randomized clinical trials that demonstrate a mortality benefit for LCS, and it is thought that surgical resection of early-stage cancers is associated with the demonstrated mortality benefit of screening.^2,3,9^ Although stereotactic body radiotherapy is used for inoperable early-stage cancers, often for individuals who are poor surgical candidates, we know of no published randomized clinical trial that has been able to recruit enough patients to adequately compare the benefit of these 2 approaches.^38^ It may be that nonsurgical treatment such as stereotactic body radiotherapy, given before the onset of signs or symptoms of lung cancer, provides a mortality benefit even for those in frail health. Alternatively, competing causes of death such as cardiovascular and/or pulmonary disease may overwhelm any benefit of early treatment of lung cancer. Individuals with stage I lung cancer who were healthy enough to receive surgery in national registry data were 41% more likely to die for any reason than NLST participants with stage I disease^9^; if NLST participants were compared with those who were too ill for surgery, the difference in all-cause mortality would be even larger.

In general, the use of tests and treatments of unknown or limited benefit is widespread in the US health care system and is associated with health care costs and harms.^39,40^ However, LCS has been largely absent from this discussion, perhaps because of its recency or because of the absolute low uptake of LCS, which was observed in this study and others. Efforts are certainly needed to increase outreach to LCS-eligible individuals, who may be particularly difficult to reach with preventive health interventions,^41,42,43^ while also minimizing guideline-discordant screening among individuals in poor health. In the future, eligibility criteria based on individualized risk may mitigate this challenge to some degree.^44^Substantial efforts are under way to increase LCS rates,^45^ which is a priority for Healthy People 2030,^46^ yet prior experience with other forms of cancer screening^47^ and the results presented here suggest that LCS is likely to be overused among those in frail health unless there is a more concerted effort to emphasize health status in initial screening decisions.^48^

This large population-based study corroborates findings from single centers of racial disparities in LCS rates.^16,17,18,19^ Prior analyses of BRFSS data to investigate racial or ethnic disparities have been limited by low numbers of respondents who are not non-Hispanic White^35^ and lack of adjustment for health status.^20^ LCS may offer a greater mortality benefit for non-Hispanic Black individuals than non-Hispanic White individuals,^15^ and non-Hispanic Black individuals are at higher risk of dying from lung cancer.^49^ Therefore, the racial inequalities in screening rates observed in this study and others^16,17,18,19^ as well as those in terms of LCS eligibility^20^ and adherence^50,51,52^ merit urgent attention. Recent changes to US Preventive Services Task Force eligibility criteria to lower the age and pack-year history of screening eligibility (from 55 to 50 years, and from 30 to 20 pack-years, respectively) are in part intended to equalize screening access across racial and ethnic groups, as non-Hispanic Black individuals experience higher rates of lung cancer than non-Hispanic White individuals with the same cigarette usage and same age.^53,54^ However, changing eligibility requirements alone will not fix the observed disparities in patterns of use. Widespread implementation of LCS will not achieve its maximal impact without understanding and addressing reasons for these disparities.

### Strengths and Limitations

Strengths of the study include that data were drawn from a population-based sample with a standardized assessment of smoking history to ascertain LCS eligibility; self-reported smoking history is reliable^55^ and often unavailable from the electronic medical record.^56^ Self-rated health is strongly associated with mortality,^57^ performing more accurately than a 30-item objective measure of health and functional status in estimating 5- and 10-year all-cause mortality.^58^ Furthermore, survey data on self-rated health perform similarly across different racial and ethnic groups in the US relative to objective measures of health.^59^ Self-rated health status along with the ability to climb stairs was selected by the NELSON trial investigators as a key metric to gauge baseline health.^10^ Furthermore, our sensitivity analyses, which relied on objective comorbidities and functional limitations, provided consistent results.

This study has multiple limitations that deserve comment. Demographics, health status, and receipt of LCS were all self-reported; the validity of self-reported LCS has not yet been quantified, to our knowledge, but would likely be similar across health and racial and ethnic categories as LCS is not a particularly stigmatized or desired behavior. Thus, misclassification of screening status would tend to bias our results toward the null, if present. A minority of BRFSS respondents identified as non-Hispanic Black (3%, unweighted), which likely limits the generalizability of our findings and highlights the need to respectfully engage racial and ethnic minority groups in future LCS research. We could not directly ascertain whether a given individual was willing and able to undergo lung surgery; this is a limitation of all publicly available data sets^60^ and, indeed, routine health data, as clinicians have no recommended tool to systematically assess health status prior to ordering LCS. Although evaluation before thoracic surgery includes well-established metrics such as forced expiratory volume in 1 second,^61^ it would be infeasible to require formal pulmonary function tests or other objective diagnostic studies for all individuals considering LCS. Functional ability is a commonly used preoperative measure of cardiovascular fitness.^62^ We could only include diagnoses ascertained by BRFSS; a notable exception is hypertension, which is assessed only in odd survey years.^63^ Residual confounding could be present; if so, the true association between non-Hispanic Black individuals and LCS would be even lower than observed (ie, aOR even further from 1, meaning larger racial or ethnic disparities). The cross-sectional nature of the survey precludes inferences about the time sequence of reported associations; however, as the survey only asked about LCS in the prior 12 months, many reported comorbidities likely predated receipt of LCS. Similar to prior studies,^12,13,20,21,22,35,36,64,65^ we were unable to assess whether scans reported by respondents were truly screening tests or ordered for diagnostic or surveillance reasons. This is a major limitation of using publicly available or routine data to study LCS and must be addressed in future research to validate these findings.

## Conclusions

This population-based study of US adults in 28 states shows that implementation of LCS has resulted in a mismatch between those who have undergone screening and those who are most likely to benefit from it. Many US adults who have been screened may not accrue its benefits because of underlying poor health, and although non-Hispanic Black individuals may experience a greater mortality reduction from screening, they are less likely than non-Hispanic White individuals to undergo lung cancer screening. There is an opportunity at this fairly early stage of LCS implementation to improve concordance between who is recommended to receive screening and who is actually screened to reach those individuals who are most likely to benefit.

## Notes

Supplement. **eFigure.** Prevalence of Specific Functional Limitations and Comorbidities by Self-Reported Health Status, Among 14 550 Individuals Representing 3.68 Million Lung Cancer Screening-Eligible US Residents, 2017 to 2020
