# Errors in Abstract, Introduction, and Methods

**DOI:** 10.1001/jamanetworkopen.2022.13694

**Published:** 2022-05-06

In the Original Investigation titled “Likelihood of Lung Cancer Screening by Poor Health Status and Race and Ethnicity in US Adults, 2017 to 2020,”^1^ published March 31, 2022, there were errors in the Abstract, Introduction, and Methods. These sections should have stated that participants had a greater than or equal to 30 pack-year smoking history. This article has been corrected.^1^
