# Supplementary material for: Likelihood of Lung Cancer Screening by Poor Health Status and Race and Ethnicity in US Adults, 2017 to 2020
Source: JAMA Netw Open. 2022 Mar 31;5(3):e225318. doi: 10.1001/jamanetworkopen.2022.5318 (PMC8972038; doi:10.1001/jamanetworkopen.2022.5318)
Supplement: Supplement. — eFigure. Prevalence of Specific Functional Limitations and Comorbidities by Self-Reported Health Status, Among 14 550 Individuals Representing 3.68 Million Lung Cancer Screening-Eligible US Residents, 2017 to 2020 [file jamanetwopen-e225318-s001.pdf]

Supplemental Online Content

Rustagi AS, Byers AL, Keyhani S. Likelihood of lung cancer screening by poor health status and race and ethnicity in US adults, 2017 to 2020. *JAMA Netw Open*. 2022;5(3):e225318. doi:10.1001/jamanetworkopen.2022.5318

eFigure. Prevalence of Specific Functional Limitations and Comorbidities by Self-Reported Health Status, Among 14 550 Individuals Representing 3.68 Million Lung Cancer Screening-Eligible US Residents, 2017 to 2020

This supplemental material has been provided by the authors to give readers additional information about their work.

© 2022 Rustagi AS et al. *JAMA Network Open.*

eFigure. Prevalence of specific functional limitations and comorbidities by self-reported health status, among n=14,550 individuals representing 3.68 million lung cancer screening-eligible US residents, 2017-2020. For all associations, p<0.001 for linear trend.

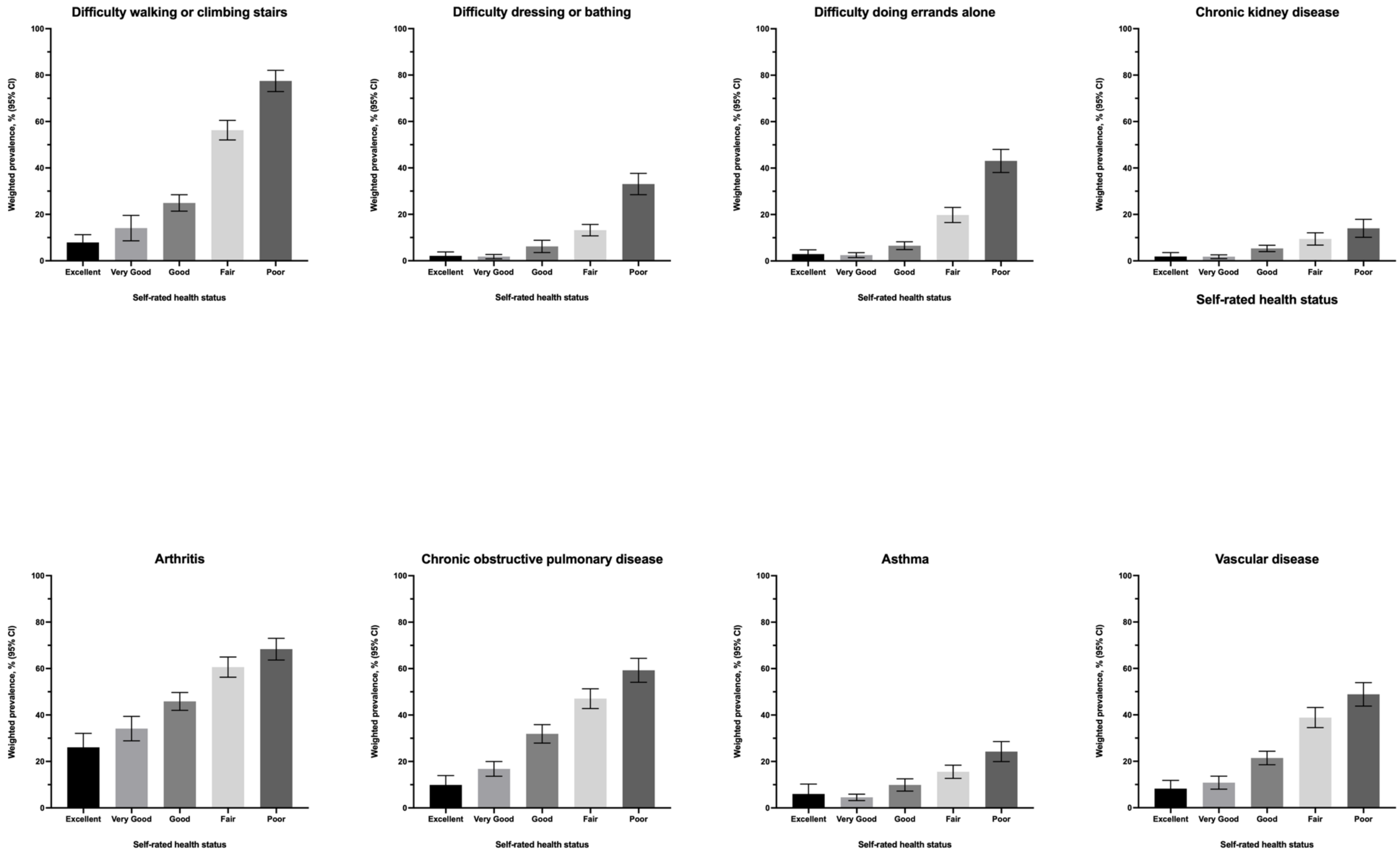

© 2022 Rustagi AS et al. *JAMA Network Open.*
